# Efficacy of a smoking cessation program in a population of adolescent smokers in vocational schools: a public health evaluative controlled study

**DOI:** 10.1186/1471-2458-13-149

**Published:** 2013-02-18

**Authors:** Laetitia Minary, Linda Cambon, Hervé Martini, Nathalie Wirth, Dovi S Acouetey, Francine Thouvenot, Céline Maire, Yves Martinet, Abraham Bohadana, Denis Zmirou-Navier, François Alla

**Affiliations:** 1 INSERM, CIC-EC, CIE6, Nancy, F-54 000, France; 2 CHU Nancy, Epidémiologie et Evaluation Cliniques, Nancy, F-54 000, France; 3 Université de Lorraine, Université Paris Descartes, Apemac, EA 4360, Nancy, F-54 000, France; 4 INSERM U 954, Faculté de Médecine, Nancy, France; 5 IREPS Lorraine, Nancy, France; 6 Réseau Lorrain d’Alcoologie et des Dépendances Associées, Hôpital Villemin, Centre Hospitalier Universitaire Nancy, Nancy, France; 7 CHU de Nancy, Service de pneumologie, Nancy, France; 8 Epinal, France; 9 EHESP School of Public Health, Rennes, France

**Keywords:** Tobacco cessation, Evaluation, Adolescent, Addiction, Smoking prevention & control

## Abstract

**Background:**

To evaluate the public health efficacy of a community-based smoking cessation program (TABADO) among vocational school trainees (15 to 20 years old).

**Methods:**

This prospective, controlled, quasi-experimental study was conducted in eight vocational training centres (VTC) in France. The intervention group underwent the TABADO program, which included a general information session for all students and small-group sessions plus individual counselling and nicotine therapy, if needed, for volunteers in an enhanced program. The control group received no specific intervention other than the educational services usually available. The primary outcome was 30-day point prevalence abstinence at 12 months.

**Results:**

The mean age of the 1,814 students included was 16.9 years (SD = 1.0); 84.7% were males. At baseline, 52% were smokers and 5.7% ex-smokers. In the intervention group, 24.6% of smokers volunteered for the enhanced program and 18.1% could be included. By 12-month follow-up, with participants lost to follow-up considered non-abstinent, 10.6% of smokers in the intervention group had become abstinent versus 7.4% in the control group (adjusted p = 0.03; odds ratio [OR] = 1.8; 95% confidence interval [CI] = 1.05–3.0); considering lost to follow-up as missing data, 17% of intervention group participants were abstinent versus 11.9% in the control group (univariate p = 0.08; adjusted p = 0.008; OR = 2.1; 95% CI = 1.2–3.6).

**Conclusion:**

The TABADO program, targeting teenagers in vocational schools, was effective in producing a higher 12-month abstinence rate among all smokers in the intervention group.

**Trial registration:**

Clinical trial identification number is NTC00973570.

## Background

Most smoking prevention initiatives undertaken in the adolescent population focus on preventing smoking initiation. Considering that most teenagers have already experimented smoking, and that dependence occurs very early even in occasional smokers [1], it is also important to help these adolescents quit with an adapted smoking cessation program.

Programs to help adolescent smokers are many. Nine reviews and meta-analyses have been conducted on this subject [2-10]. They concluded to the effectiveness of cognitive-behavioral strategies, but highlighted the lack of evidence regarding results of pharmacological strategies. Moreover, the success of a smoking cessation program depends on a support strategy for smokers but also other factors [4,8,9] as: the manner to deliver first lecture (informative but not preachy), the accessibility of treatment programs (geographical by implementing them within schools -integrating the programs during school hours- and financial with their cost-free character- for consultation and nicotine replacement substances), the anonymity of the program and the voluntary inclusion in the program.

A smoking cessation program called TABADO, based on these elements was developed by a multidisciplinary team in Nancy, France [11]. This community-based program combines information sessions for all smokers and non-smokers, as well as medication and cognitive behavioural therapy for smokers who volunteer for the enhanced program.

This program was designed to target a particularly vulnerable population, vocational school trainees, since 49.9% of 17-year-old French students in apprenticeships are daily smokers, versus 28.9% in the general population of the same age [12].

This evaluation study was conducted in vocational training centres (VTCs) in the Lorraine region of France. That study’s design has been described in detail elsewhere [13]. The main objective of this study was to evaluate the efficacy of offering this community-based intervention as part of a comprehensive approach to prevention in a population of young trainees in VTCs. From a public health perspective, the unit of intervention is the community [14].

## Methods

### Design

This controlled, prospective, quasi-experimental study compared two groups. The intervention group underwent the TABADO program, and the control group, drawn from the same training curriculum but from different VTCs, received no specific intervention other than the educational services usually available.

### Setting and participants

The sampling pool for the intervention and control groups included all students attending a participating VTC in the Lorraine region (eastern France, 2.3 million inhabitants, 51 VTCs, 16,500 trainees) during the school years 2007–2008 and 2008–2009. For logistical reasons, we included only classes of more than 10 pupils whose training schedule (i.e., one week of VTC courses alternating with three weeks at their employer’s facility) was similar.

All VTCs in Lorraine (n = 51) were invited to participate. Among them, eight agreed to be included in the study and to be designated indiscriminately as either an intervention or a control school. Because of the limited number of centres and their strong dissimilarities in terms of training courses and size (from 30 to 300 apprentices), we used pragmatic sampling rather than randomization to allocate the centres (each was assigned to either the intervention or control group at the time of its inclusion with a view to ensuring students’ areas of study were balanced between both groups).

#### Inclusion criteria

All students, male and female, 15 to 20 years old inclusively, who were registered in the participating VTCs for a two-year training course were included in the sample pool. Information session on tobacco consumption was delivered to all participants regardless of smoking status. Excluded from participation were students who had current serious psychiatric disorders or who were at risk of psychological problems on quitting smoking (major depression) or who were already involved in an ongoing attempt to quit with medical monitoring.

Ethical approval for the trial was received from INSERM (the National Health and Medical Research Institute in France). The protocol was submitted to the appropriate national scientific and ethical bodies (CCTIRS and CNIL), who gave their approval. Written consent was obtained from the participants in the enhanced program, after they were given information about the study. For volunteers under 18 years old, consent also had to be obtained from their legal representatives (parents or guardian), with the result that some (n = 18) decided not to participate because they did not want to engage in a discussion with their parents.

### Intervention

The TABADO intervention combined a general program aimed at all participants, in the form of an information session on tobacco consumption, with a specific *enhanced program* undertaken by some smokers on a volunteer basis (identified here as “EP participants”) [11]. This volunteer contingent underwent two stages: 1) individual consultation with a team of physicians specialized in tobacco addiction who visited the VTC and provided personalized assistance with choosing nicotine replacement therapy (patches or gums), if needed; and 2) a small group approach, supervised by the same physicians, consisting of discussion sessions to share experiences, strengthen motivation and prevent relapse. Each group underwent four sessions spread over three months (to accommodate the 1:3 week training schedule described above); preceding those group sessions, individual counselling was also offered to each student. In cases where nicotine replacement treatment was contraindicated (hypersensitivity to one of the components, occasional smoking, or skin infection that might interfere with the patch, if used), only the four cognitive-behavioural group sessions preceded by individual counselling were provided.

### Sample size calculation

Based on the literature, we expected a maximum 5% spontaneous quit rate [15]. We hypothesized this rate would double in the intervention group’s EP participants after one year. This 10% rate was based on the assumption of an effective participation of 50% of smokers in the intervention group, with a smoking cessation rate of 15%, and the regular 5% for non-EP participants in the intervention group. With two groups of the same size, a two-sided α risk of 5% and a power of 85%, 500 smokers per group were needed. Thus, the total number of students to be included was 2000 (anticipating a smoking prevalence of 50%).

### Data collection

#### Monitoring data for all students

The program was implemented over two inclusion periods: the first beginning in February 2008, and the second in November 2008. In both cases, data collection began with a first visit, conducted within the same period for both intervention and control schools, during which all students completed an initial assessment questionnaire asking about smoking status, sociodemographic data, knowledge, attitudes and behaviour. A final questionnaire to evaluate smoking status was administered at 12 months and contained the items in the initial questionnaire plus items to assess changes in perception of health risks associated with smoking. The final questionnaire also included items to measure changes over time in tobacco consumption among smokers. Thus, it enabled an overall assessment of the efficacy of the intervention among the target population. Clinical research assistants distributed and collected the questionnaires.

The primary outcome was 30-day point prevalence abstinence at 12 months. The rate of abstinence was defined by the number of baseline smokers who had quit at 12 months relative to the total number of baseline smokers. Baseline smokers were defined as respondents who reported that they smoked at the time of the initial questionnaire (on the question: “Are you a smoker or non-smoker?”). Abstinence was defined as being a non-smoker (i.e., ex-smoker) at 12 months and having not smoked for at least one month before that point (data from two questions in the final survey: “Are you a smoker, non-smoker, or ex-smoker?” and “How long have you not been smoking?”). The validity of responses regarding smoking status in the questionnaire was ensured by measuring expired carbon monoxide concentrations in 140 students selected at random (the calculated sample size was 130, for 90% sensitivity, 5% precision and 5% alpha). Among the 69 smokers detected by analysis of expired carbon monoxide, 68 had identified themselves as smokers, yielding a questionnaire sensitivity of 98.5% (95% CI = 96.5–100).

The secondary outcome was overall prevalence of tobacco use at 12 months.

#### Monitoring data for smokers who volunteered for the enhanced program

The tobacco addiction specialists used an initial questionnaire to assess the EP participants’ tobacco consumption in depth. Their tobacco consumption was subsequently monitored over time by another questionnaire completed at each of the four individual counselling sessions.

### Statistical analysis

30-day point prevalence abstinence at 12 months was compared by multivariate logistic regression adjusting for predefined characteristics (age, sex, and training course), differing characteristics between the two groups at baseline (i.e., cannabis consumption, Hooked On Nicotine Checklist [HONC] score) and tobacco consumption.

The analysis of VTC effect by estimating the intraclass correlation coefficient(ICC) led us to decide to choose a classical model rather than a hierarchical model (intraclass correlation coefficient = 0.04). The analysis was based on intention to treat. Loss to follow-up was considered in a first analysis as non-abstinence and in a second analysis as missing data. Comparisons between two categorical variables were done by chi-square test and between two continuous variables by Student’s *t* test. A p-value of <0.05 was considered statistically significant. Analyses used SAS 9.2 (SAS Institute, Cary, NC).

## Results

**Figure 1:**
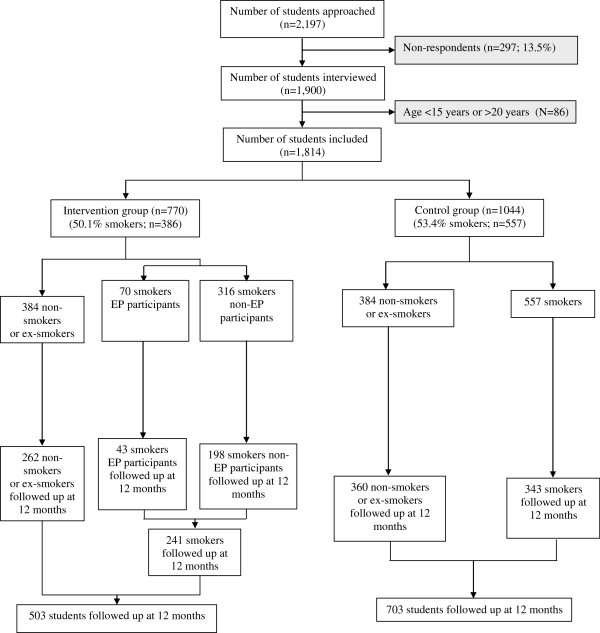
**Flow chart illustrating the inclusion and follow-up of students in the TABADO study.** TABADO study, Nancy, France, 2008-2009.

Three VTCs were selected as intervention sites and five as controls. Of the 2,197 students in these VTCs who were in a two-year program and in classes with more than 10 students, 297 were either absent on the day of the visit by the research assistant administering the questionnaire or chose not to respond to the questionnaire. Thus, 1,900 students completed the baseline questionnaire, 86 of whom were below 15 or above 20 years old. In the end, 1,814 (82.4%) questionnaires were usable (Figure 1). Of these students, 770 were included in the intervention group and 1,044 in the control group. The mean age of students was 16.9 (SD = 1.0) years, and 84.7% were males (81.8% in the intervention group and 87.0% in the control group). Students were involved in three areas of vocational learning: building and public works (64.0%), the catering industry (22.7%) and personal services (13.3%). Of the 1814 students interviewed, 52.0% were smokers, 42.3% non-smokers and 5.7% ex-smokers. Thus, the study population for the primary objective comprised 943 smokers, representing 50.1% (n = 386) of the intervention group and 53.4% (n = 557) of the control group (Table 1).

**Table 1:** Comparison of baseline characteristics of students in the intervention and control groups

	**Intervention**				**Control**				
	**N = 770 (42.4%)**				**N = 1,044 (57.6%)**				
	**N**	**%**	**μ**	SD*	**N**	**%**	**μ**	SD*	p**
Age	735		16.8	1.0	998		16.9	1.0	0.03
Sex									0.002
Male	630	81.8			908	87.0			
Female	140	18.2			136	13.0			
Area of training									0.09
BPW^£^	472	62.3			681	65.6			
Catering industry	171	22.6			236	22.7			
Personal services	115	15.2			121	11.7			
Smoking status									0.25
Non-smoker	334	43.4			434	41.6			
Smoker	386	50.1			557	53.4			
Ex-smoker	50	6.5			53	5.1			

^£^ BPW = building and public works.

*SD = standard deviation **p-value <0.05. Data are mean ± SD. BPW: building and public works.

Smokers in the two groups were comparable (Table 2), except for sex (80.3% males in the intervention group versus 88% in the control group, p = 0.001) and dependence score (HONC score; 6.4 ± 2.7 in the intervention group versus 5.9 ± 2.9 in the control group, p = 0.01). Also, students in the control group more frequently reported smoking or having smoked cannabis (p = 0.03).

**Table 2:** Comparison of baseline characteristics of smokers in the intervention and control groups

	**Intervention**				**Control**				
	**N = 386 (40.9%)**				**N = 557 (59.1%)**				
	**N**	**%**	**μ**	SD*	**N**	**%**	**μ**	SD*	p**
*Sociodemographic variables*									
Age	364		16.9	1.0			17.0	1.0	0.16
Sex									0.001
Male	310	80.3			490	88.0			
Female	76	19.7			67	12.0			
Area of learning									0.09
BPW^£^	472	62.3			681	65.6			
Catering industry	171	22.6			236	22.7			
Personal services	115	15.2			121	11.7			
Cannabis consumption	203	54.3			334	62.2			0.02
*Smoking behaviour*									
Age at first cigarette	346		12.1	2.1	514		12.2	2.1	0.59
Age at which smoking became a daily occurrence	324		13.7	1.6	488		13.9	1.7	0.25
Current number of cigarettes/day	365		13.0	8.5	536		12.7	7.3	0.62
Motivation for quitting smoking (scale: 0 to 10)	171		3.2	3.1	280		3.4	3.0	0.53
Chances of succeeding (scale: 0 to 10)	170		4.3	3.1	278		4.8	3.2	0.16
HONC score	351		6.4	2.7	538		5.9	2.9	0.01
Type of smoking behaviour									0,38
Casual	21	12.2			27	9.6			
Daily	151	87.8			254	90.4			
Heavy smokers (>10 cig/d)	174	48.6			255	48.5			0.97
Number of attempts to quit smoking									0.50
Never	106	39.7			146	36.8			
Once	77	28.8			119	30.0			
2 to 3 times	72	27.0			101	25.4			
4 to 5 times	8	3.0			19	4.8			
More than 5 times	4	1.5			12	3.0			
Physician consultations about quitting smoking	3	1.7			6	2.1			0.77
Use of a nicotine substitute to quit smoking	11	6.4			22	7.9			0.55
Intention to quit smoking within the next six months	80	30.7			109	29.5			0.75

*SD = standard deviation **p-value <0.05.

Data are mean ± SD.

^£^ BPW: building and public works; HONC: Hooked On Nicotine Checklist.

### Participation in the enhanced program

Of the 386 students who were smokers in the intervention group, 95 expressed a desire to participate in the enhanced program (EP) (24.6%) and 70 of those were included (18.1%). The 25 others presented non-inclusion criteria: 18 did not have parental consent and seven had medical contraindications (e.g. major depression). The mean baseline HONC score was higher for EP participants compared to non EP participants (7.1 vs. 6.2; p = 0.02 – Table 3), as were intention to quit within six months (55.6% vs. 26.5%; p = 0.0004) and daily smoking (100% vs. 85.8%, p = 0.03). Sex and area of learning also differed between groups. These variables were identified as adjustment covariates in multivariate analysis.

**Table 3:** Comparison between EP participants and non-EP participants smokers

	**Non EP participants**				**EP participants**				
	**N=316 (81.9%)**				**N=70 (18.1%)**				
	**N**	**%**	**μ**	SD*	**N**	**%**	**μ**	SD*	p**
*Sociodemographic variables*									
Age	299		16,9	1,0	66		17,1	1,0	0,18
Sex									0,02
Male	248	78,2			63	90,0			
Female	69	21,8			7	10,0			
Area of learning									0,01
BPW^£^	195	62,3			56	81,2			
Catering industry	61	19,5			6	8,7			
Personal services	57	18,2			7	10,1			
Cannabis consumption	160	52,3			43	63,2			0,10
*Smoking behaviour*									
Age at first cigarette	284		12,1	2,0	60		12,0	2,1	0,68
Age at which smoking became a daily occurrence	262		13,7	1,6	60		13,7	1,7	0,97
Current number of cigarettes/day	294		12,9	8,8	69		13,7	6,6	0,51
Motivation for quitting smoking (scale: 0 to 10)	147		3,1	2,9	24		4,0	3,9	0,19
Chances of succeeding (scale: 0 to 10)	146		4,2	3,2	24		5,0	2,7	0,27
HONC score	283		6,2	2,8	66		7,1	2,5	0,02
Type of smoking behaviour									0,03
Daily	127	85,8			24	100			
Casual	21	14,2							
Heavy smokers (>10 cig/d)		46,2			40	58,8			0,06
Number of attempts to quit smoking									0.50
Never	97	42,9			9	23,1			
Once	65	28,8			12	30,8			
2 to 3 times	53	23,5			17	43,6			
4 to 5 times	7	3,1			1	2,6			
More than 5 times	4	1,8							
Physician consultations about quitting smoking	2	1,3			1	4,2			0,32
Use of a nicotine substitute to quit smoking	8	5,4			3	12,5			0,19
Intention to quit smoking within the next six months	58	26,5			21	55,6			0,04

* Standard Deviation ** Chi-2 for qualitative variables, Student’s t test for quantitative variables.

### Follow-up of students

Of the 1,814 students in the study (Figure 1), 1,206 were questioned again at 12 months in both categories of VTCs (66.5%: 65.3% in the intervention group and 67.3% in the control group). The proportion of males was higher in non-respondents than in respondents (88.5% vs*.* 83.0%, p = 0.002), and non-respondents were older (mean 17.0 ± 1.1 vs*.* 16.8 ± 0.9 years, p <0.0001).

### Primary assessment criteria

Among the baseline smokers, and considering those lost to follow-up as non-abstinent, 10.6% in the intervention group were abstinent at 12 months versus 7.4% in the control group (univariate p = 0.08; adjusted p (for age, sex, training course, initial cannabis consumption, HONC score, smoking consumption) = 0.03; OR 1.8; 95% CI = 1.05–3.0).

Among the baseline smokers, and considering those lost to follow-up as missing data, 17% in the intervention group were abstinent at 12 months versus 11.9% of smokers in the control group (univariate p = 0.08; adjusted p = 0.008; OR 2.1; 95% CI = 1.2–3.6) (Table 4).

**Table 4:** Rate of smoking abstinence in the intervention and control groups at 12 months

	**Abstinence rate**		**OR unadjusted**	**p**	**OR adjusted**	** p**
	**Intervention group (N = 41)**	**Control group (N = 41)**				
Smokers (LFU = smokers at 12 months)	10.6%	7.4%	1.5 [0.95–2.3]	0.08	1.8 [1.05–3.0]	0.03
Smokers (LFU = missing data)	17%	11.9%	1.5 [0.94–2.4]	0.08	2.1 [1.2–3.6]	0.008

p-value <0.05 *univariate **for age, sex, training course, initial cannabis consumption, HONC score, smoking consumption.

LFU: Lost to follow-up; OR: odds ratio; 95% CI: 95% confidence interval.

In the intervention group (Table 5), the abstinence rate among EP participants was 5,7% versus 11.7% among non-EP participants (univariate p = 0.21; adjusted p = 0.37). There was no statistical difference between the 2 subgroups.

**Table 5:** Rate of smoking abstinence among EP participants and non-EP participants at 12 months

	**Abstinence rate**		**OR**	**p**	OR**	** p**
	**EP participants (N =4)**	**Non-EP participants (N = 37)**				
Smokers (LFU = smokers at 12 months)	5.7%	11.7%	0.5 [0.2–1.5]	0.21	0.5 [0.1–2.1]	0.37
Smokers (LFU = missing data)	9.3%	18.4%	0.5 [0.2–1.5]	0.20	0.6 [0.1–2.5]	0.47

p-value <0.05 *univariate **for age, sex, training course, initial cannabis consumption, HONC score, smoking consumption.

LFU: Lost to follow-up; OR: odds ratio; 95% CI: 95% confidence interval.

Of the 70 EP participants, 33 (47.1%) received nicotine replacement therapy (NRT) (patch or gum) and used it. At 12 months, 11.5% of the NRT users were abstinent versus 5.6% among EP participants who didn’t needed NRT according to physician or/and student.

### Secondary outcome

At 12 months, smoking prevalence was 50.9% (+2.1%) for the control group and 48.9% (+1%) for the intervention group (for evolution: crude p = 0.76; adjusted p = 0.75).

### Group effect

To study this effect more specifically, we conducted post hoc further analysis on the abstinence rate among non-EP participants in each class based on the number of EP participants in that class. The abstinence rate by class differed significantly depending on the number of EP participants: the abstinence rate among non-EP participants was 15.2% when there were fewer than two EP participants in the classroom versus 25.4% when there were at least two EP participants (p = 0.04 after adjustment for age, sex, baseline dependence and cannabis use).

## Discussion

This study of the provision of the TABADO smoking cessation program to trainees in VTCs found a higher smoking cessation rate at 12 months in the intervention group. After adjustment, an odds ratio of 1.8 indicated that smoking cessation occurred almost twice as often in the intervention group than in the control group (p=0.03). Among intervention group, smoking cessation rate do not differ between EP and non-EP smokers.

Interestingly, the difference in abstinence rates observed between groups is mainly due to other students rather than to the EP participants. This may illustrate the importance of the “group effect” on smoking behaviour and on motivation to quit smoking. Christakis emphasized that the decision to quit smoking is not taken by an individual in isolation but rather reflects choices made by groups of individuals interrelated directly and indirectly [16]. Thus, a smoker’s decision to quit induces other individuals in the same social network to also consider quitting, with some actually doing so. Christakis concluded that public health interventions of a collective type aimed at inducing smokers to quit could be more effective than individual interventions because they would promote the dissemination of health behaviours among individuals in groups. We could thus hypothesize that some adolescents’ open expression of their desire to quit smoking triggered the commitment of others. The trigger for the decision may not necessarily be one person’s successful cessation, but rather the decision to stop, or at least the attempt.

According to the literature [17-22], the utility that an individual receives from pursuing a given activity depends on the actions of the other individuals in the person’s reference or peer group. Thus, an increase in the prevalence of a given behavior at the peer level may lead to an increased probability of such behavior at the individual level. We could cite as an example Powell’s study [22] showing that moving a student from a school where no one smoked to a school where a quarter of the students were smokers increased the student’s likelihood of smoking by about 14.5 percentage points.

In this TABADO study, the significant results of the effect of the number of EP participants in a classroom on the abstinence rate for non-EP participants is a strong argument in favour of our hypothesis and underscores the importance of offering a program and of assessing it on a collective rather than individual basis.

The absence of any difference in abstinence between EP participants and other smokers may be due to the EP participants’ higher dependence levels at baseline. Effectively, according to the literature, nicotine dependence is significantly associated with quitting and intention to quit among young adult daily smokers [23] but interfere with adolescents’ abilities to quit smoking [24]. Thus volunteers are more motivated what could be associated to a success’s factor but they are equally more dependant and tend to be more co-addicted. It has been largely demonstrated that co-addiction is a major barrier to quitting smoking [25]. This dual opposite effect probably causes the absence of differences between EP participants and non EP participants.

### Strengths and limitations

#### Design

This was a controlled study with a prospective longitudinal design. Thus, the smoking cessation rate in the control group was also measured, and the increase in smoking cessation in the intervention group could not be attributed to social, environmental or contextual factors such as a smoking ban. From a methodological standpoint, the observed group effect justifies our ascribing the intervention to a group rather than to an individual. From a public health perspective, it makes sense to measure the primary assessment criteria in all smokers, whether or not they participated in the enhanced program, because the school collectivity is the unit of the preventive action. We used a quasi-experimental design rather than a randomized approach, for a practical reason: the number of participating institutions was limited (n = 8) and they were strongly dissimilar in terms of training courses and size, such that randomization would probably have been ineffective [26]. Even so, despite the absence of randomization, the control and intervention groups were similar in terms of motivation, attempts and intention to quit smoking. The major differences were higher cannabis consumption in the control group (62.2% vs. 54.3%, p = 0.02) and higher dependence in the intervention group (6.4 vs. 5.9, p = 0.01). Since both characteristics are major factors of dependence, they were considered as adjustment factors.

#### Primary outcome

Most adolescent smokers who try to quit relapse within a year [27]. Hence, a 12-month follow-up is appropriate to evaluate the efficacy of the TABADO program [28].

Smoking status was evaluated by self-administered questionnaires and therefore was a self-reported statement of smoking. However, smoking status was confirmed in 140 randomly selected students by an exhaled carbon monoxide test. The questionnaire’s sensitivity was 98.5%, which demonstrated that smokers responded honestly to the question concerning their smoking status.

#### Secondary outcome

Smoking prevalence increased similarly in the two groups (+1% in the intervention group vs. +2.1% in the control group; p = 0.75). The sample size had not been calculated to assess differences in prevalences of smoking initiation.

The spontaneous abstinence rate in the general population of adolescents was previously estimated in the literature at about 5% [15]; it reached up to 7.4% in this study’s control group. This higher rate may be explained by the influence of the smoking ban in public places initiated in 2008 in France and implemented during the study period [29,30].

#### Loss to follow-up

Of the 1,814 students included in the study, 1,206 (66.5%) were followed over 12 months. Unlike adolescents enrolled in the normal school system, students attending VTCs are constantly on the move, and the number of broken vocational training contracts is high (26%) [31] which accounts for the large proportion of students lost to follow-up (33.5%). That students were not followed over 12 months did not indicate their desire to exit the study, but was rather a consequence of natural fluctuations in this population. In considering this high proportion of students lost to follow-up as still being smokers, our analysis was built upon the most adverse conditions. Yet even with a hypothesis of maximum bias—that is, even assuming all smokers lost to follow-up remained smokers—the program’s efficacy remains significant (OR 1.8; 95% CI = 1.05–3.0).

## Conclusions

Trainees at VTCs are particularly prone to being smokers. Among the smokers included in the TABADO program, almost one-quarter wished to participate in the enhanced smoking cessation program. Faced with such an expectation among adolescents for help, it is important to improve accessibility to such programs. This health promotion project, conducted using a community approach in a vocational institution, demonstrated positive effects, with health behaviour changes among adolescents.

## Abbreviations

CHU: Centre hospitalier universitaire (university hospital); CNIL: Commission nationale de l’informatique et des libertés; EP: Enhanced Program; ESCAPAD: Enquête sur la santé et les consommations lors de l’appel de préparation à la défense; ESPAD: European School Survey Project on Alcohol and Other Drugs; HONC: Hooked On Nicotine Checklist; CI: Confidence interval; INCA: Institut national du cancer; MILDT: Mission interministérielle de lutte contre la drogue et la toxicomanie; OR: Odds ratio.

## Competing interest

The authors declare they have no conflicts of interest and that the funding sources (Conseil régional de Lorraine, Institut national du cancer, Institut national de la santé et de la recherche médicale, Ligue contre le cancer, Mission interministérielle de lutte contre la drogue et la toxicomanie, Société de pneumologie de langue française) had no input into the study design, data collection, analysis and interpretation of data, the writing of the report or the decision to submit the paper for publication.

## Authors’ contributions

LM carried out the epidemiological study, drafted the manuscript, participated in the intervention for the data collection, and performed the statistical analysis. LC participated in drafting the manuscript. AB, HM, NW and YM participated in the development of the protocol. HM, NW, FT, CM and SA carried out the program as lecturers and tobacco addiction specialists. FA and DZN designed the study and drafted the manuscript. All authors read and approved the final manuscript.

## Pre-publication history

The pre-publication history for this paper can be accessed here:

http://www.biomedcentral.com/1471-2458/13/149/prepub
